# Supplementary figures and images for: Cell competition is driven by Xrp1-mediated phosphorylation of eukaryotic initiation factor 2α
Source: PLoS Genet. 2021 Dec 6;17(12):e1009958. doi: 10.1371/journal.pgen.1009958 (PMC8675920; doi:10.1371/journal.pgen.1009958)

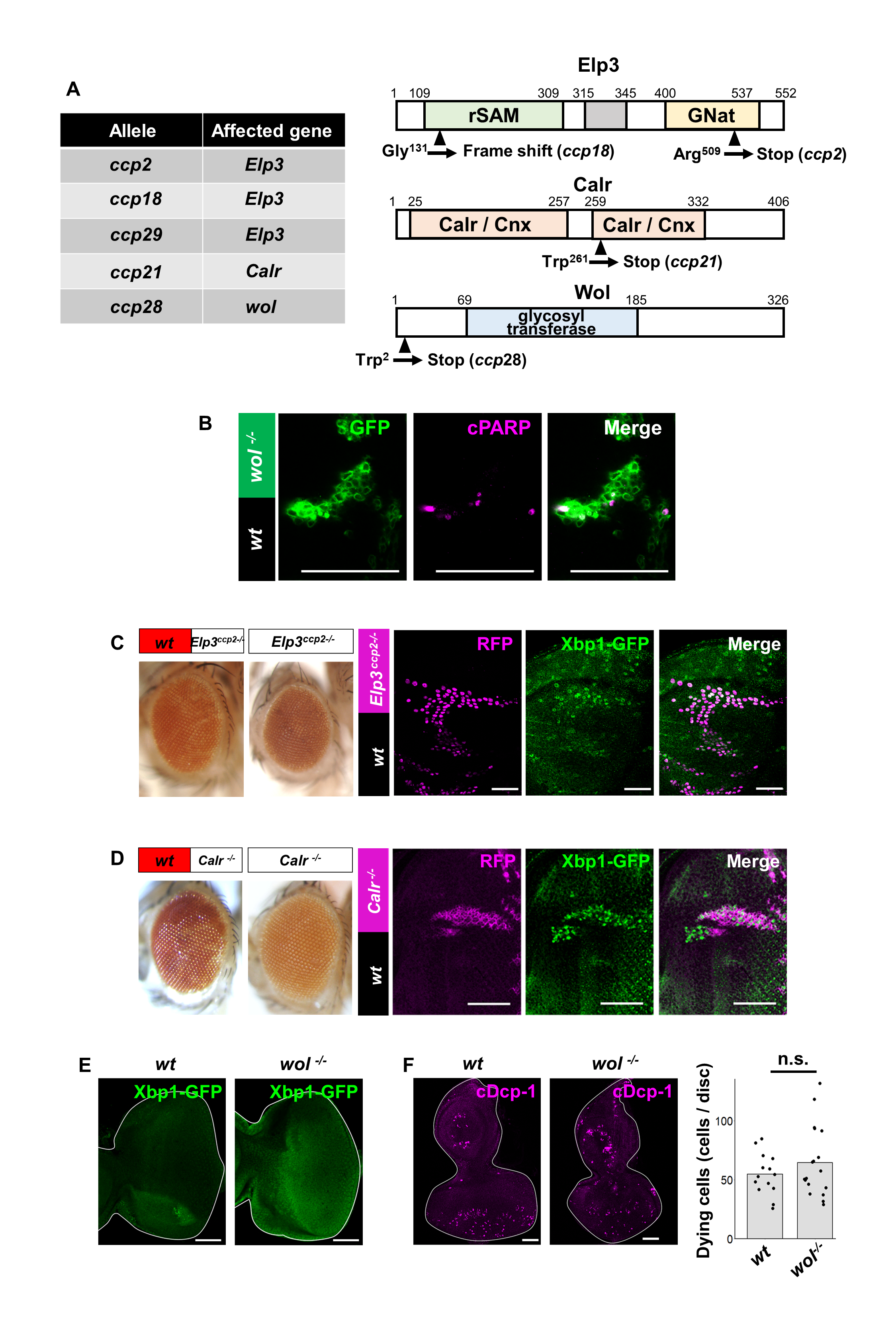

Supplement: S1 Fig — (A) A list of isolated ccp mutants that cause ER stress.and the schematic representations of the general domain structures of Elp3, Calr, and Wol, with mutations detected by the whole genome sequencing. (B) Eye disc bearing eyFLP-induced MARCM clones of mCD8-PARP-Vinus-exprssing wolccp-28-/- cells stained with anti-cleaved PARP (which detects caspase-activated dying cells). (C) Adult eye bearing eyFLP-induced mosaics of Elp3[ccp-2]-/- clones (left panel) or Elp3[ccp-2]-/- clones with surrounding wild-type tissue removed by GMR-hid and cell-lethal mutations (middle panel). Eye disc bearing eyFLP-induced MARCM clones (RFP) of Elp3[ccp-2]-/- + UAS-Xbp1-GFP cells stained with anti-GFP (right panels). (D) Adult eye bearing eyFLP-induced mosaics of Calr [ccp-21]-/- clones (left panel) or Calr [ccp-21]-/- clones with surrounding wild-type tissue removed by GMR-hid and cell-lethal mutations (middle panel). Eye disc bearing eyFLP-induced MARCM clones (RFP) of Calr [ccp-21]-/- + UAS-Xbp1-GFP cells stained with anti-GFP (right panels). Scale bars, 50μm. (E) Wild-type (left) or wolccp-28-/- (right) eye disc bearing UAS-Xbp1-GFP. In both tissues, wild-type or wolccp-28-/- clones were induced in the eye disc and then surrounding wild-type tissue was removed by GMR-hid and cell-lethal mutations. Scale bars, 50μm. (F) Wild-type (left) or wolccp-28-/- (middle) eye disc stained with anti-cleaved Dcp-1. In both tissues, wild-type or wolccp-28-/- clones were induced in the eye disc and then surrounding wild-type tissue was removed by GMR-hid and cell-lethal mutations. Scale bars, 50μm. (right) Quantification of the number of dying cells in wild-type or wolccp-28-/- eye discs. Error bars, SD; ***p<0.001 by Welch’s t-test. See S1 Text for detailed genotypes. (TIF) [file pgen.1009958.s001.tif]

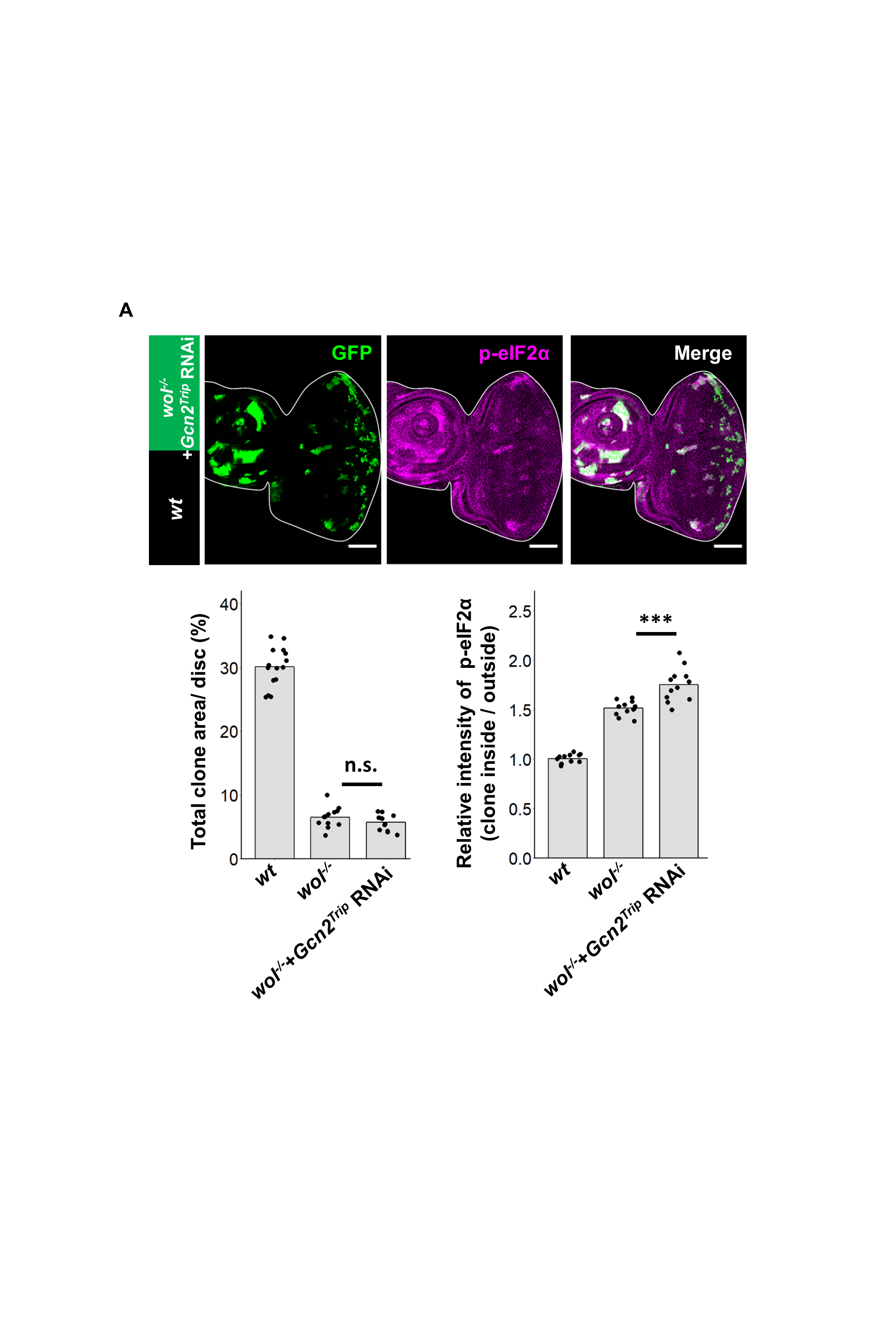

Supplement: S2 Fig — (A) Eye disc bearing eyFLP-induced MARCM clones of wolccp-28-/- + Gcn2-RNAi cells stained with anti-phosphorylated eIF2α. Quantification of the relative size of GFP-labeled clones or relative intensity of anti-phosphorylated eIF2α staining shown in A. Error bars, SD; ***p<0.001 by Welch`s t-test. Scale bars, 50μm. See S1 Text for detailed genotypes. (TIF) [file pgen.1009958.s002.tif]

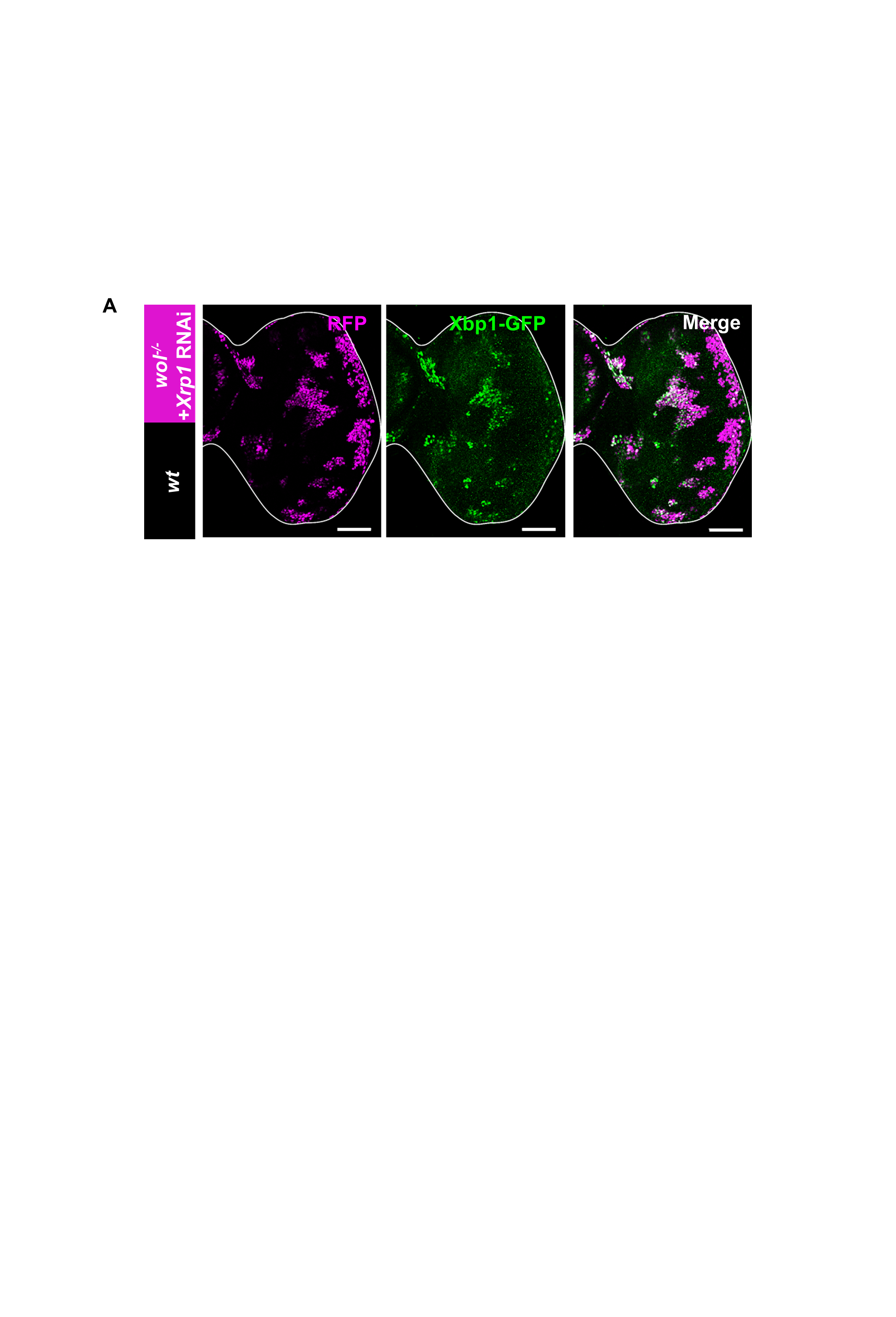

Supplement: S3 Fig — (A) Eye disc bearing eyFLP-induced MARCM clones of wolccp-28-/- + Xrp1-RNAi + UAS-Xbp1-GFP cells stained with anti-GFP. See S1 Text for detailed genotypes. (TIF) [file pgen.1009958.s003.tif]

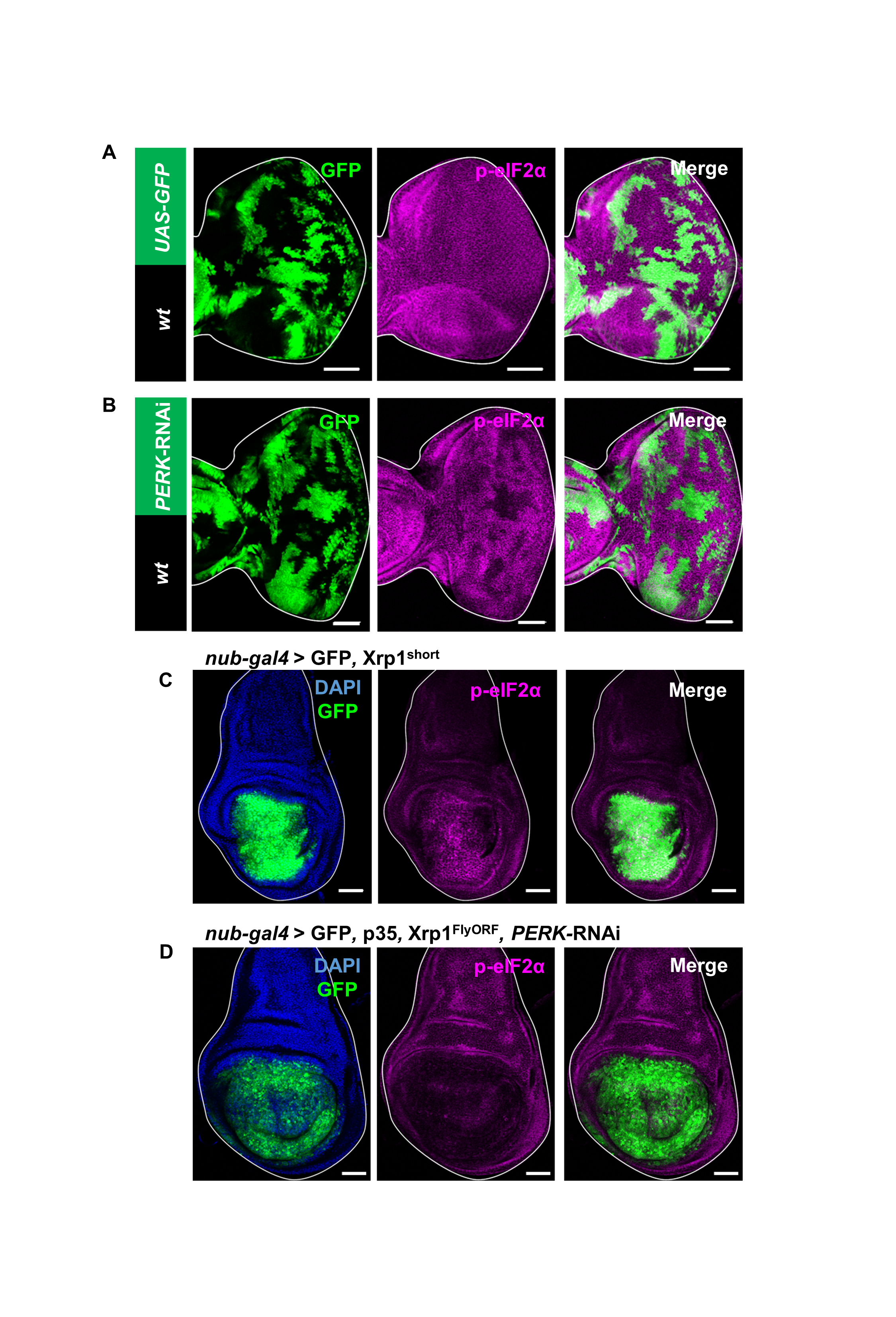

Supplement: S4 Fig — (A) Eye disc bearing eyFLP-induced MARCM clones of UAS-GFP cells stained with anti-phosphorylated eIF2α. (B) Eye disc bearing eyFLP-induced MARCM clones of PERK RNAi cells stained with anti-phosphorylated eIF2α. (C) Wing disc overexpressing GFP, Xrp1short in the wing pouch by the nub-Gal4 driver stained with anti-phosphorylated eIF2α. (D) Wing disc overexpressing GFP, Xrp1FlyORF (FlyORF: F000655) in the wing pouch by the nub-Gal4 driver stained with anti-phosphorylated eIF2α. Scale bars, 50μm. See S1 Text for detailed genotypes. (TIF) [file pgen.1009958.s004.tif]

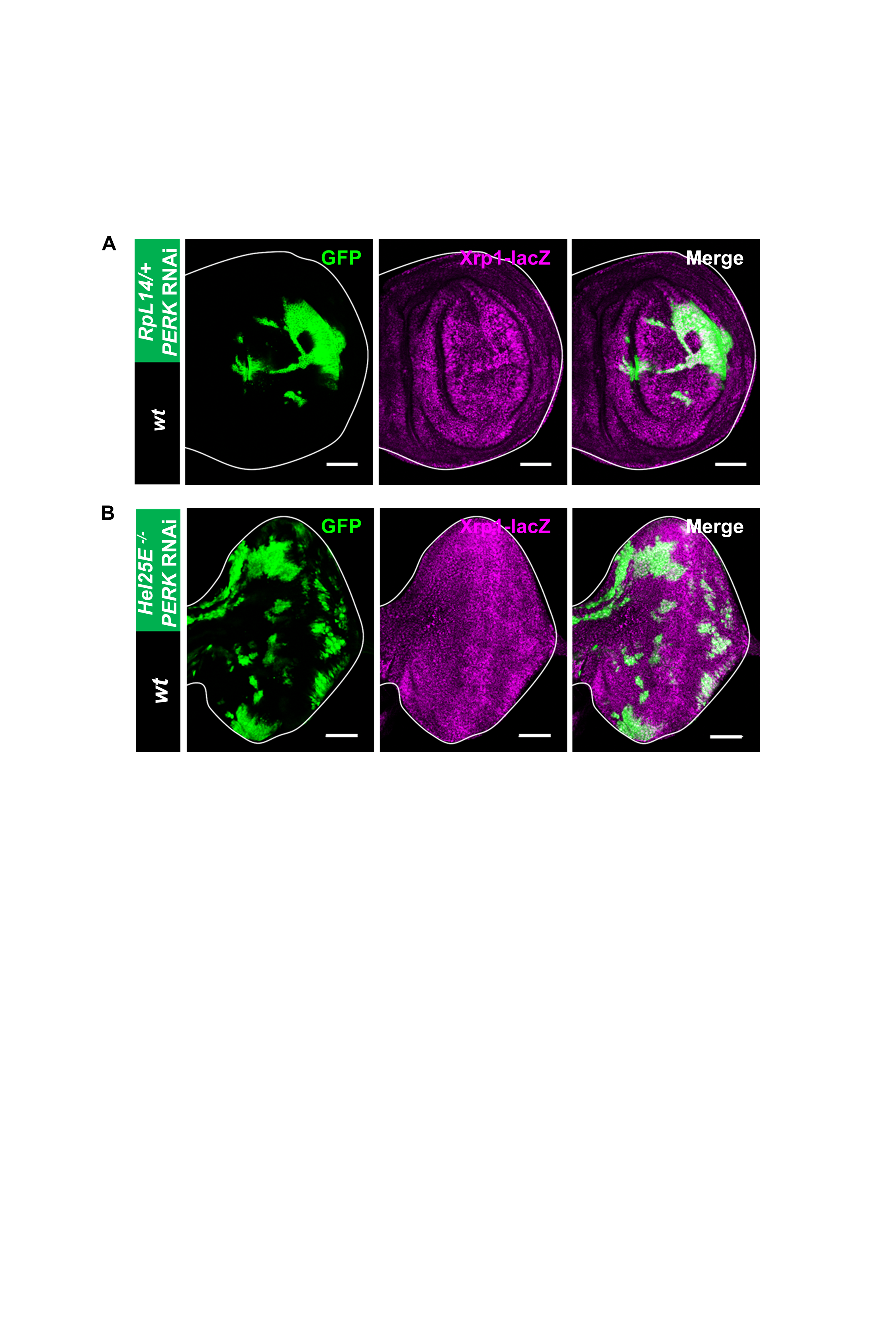

Supplement: S5 Fig — (A) Xrp1-lacZ/+ background wing disc bearing hsFLP-induced GFP-labeled clones of RpL14/+, salE>GFP + PERK-RNAi cells stained with anti-β-gal. (B) Xrp1-lacZ/+ background eye disc bearing eyFLP-induced MARCM clones of Hel25E-/- + PERK RNAi cells stained with anti-β-gal. Scale bars, 50μm. See S1 Text for detailed genotypes. (TIF) [file pgen.1009958.s005.tif]

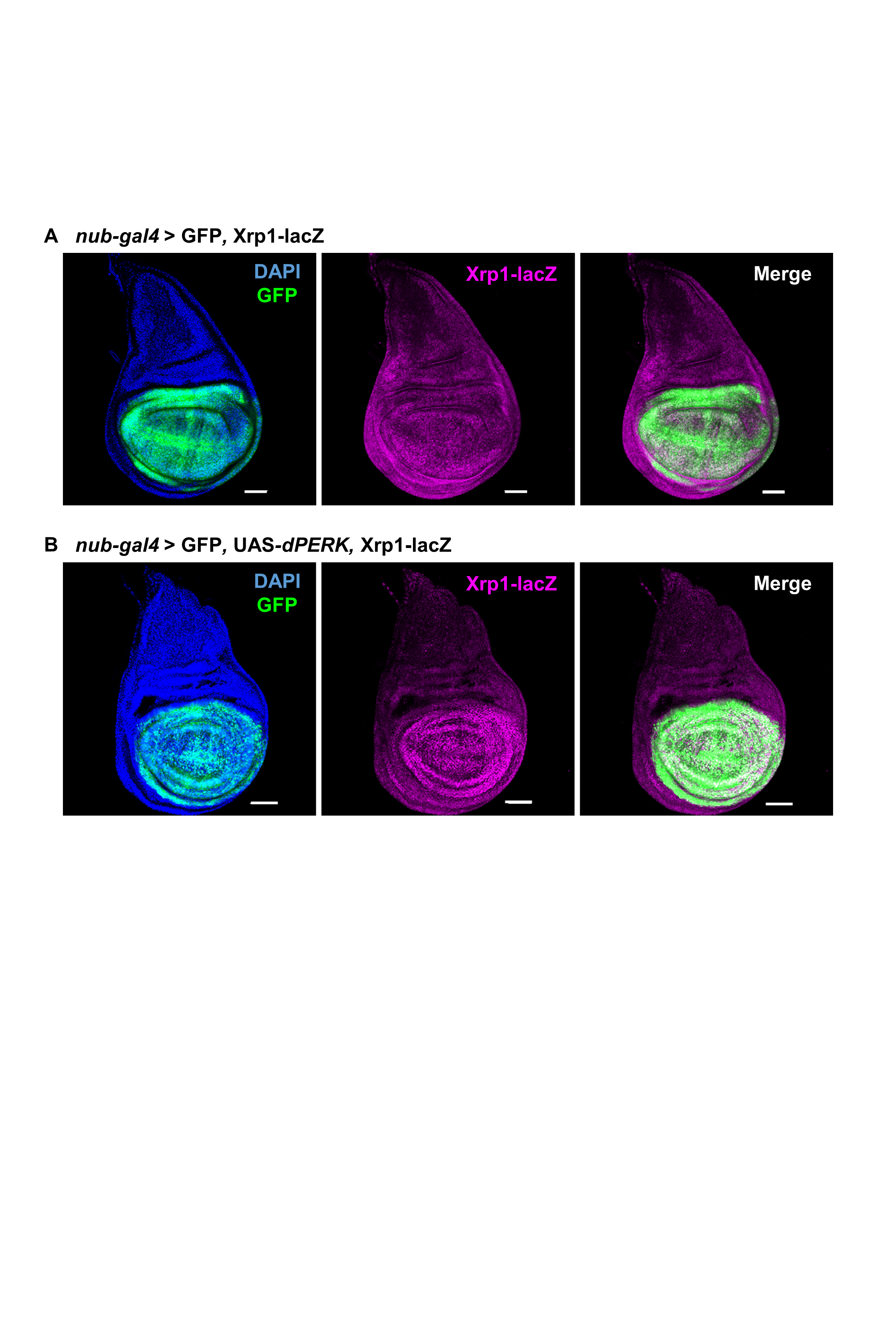

Supplement: S6 Fig — (A) Xrp1-lacZ/+ background wing disc overexpressing GFP in the wing pouch by the nub-Gal4 driver stained with anti-β-gal. (B) Xrp1-lacZ/+ background wing disc overexpressing GFP, PERK in the wing pouch by the nub-Gal4 driver stained with anti-β-gal. Scale bars, 50μm. See S1 Text for detailed genotypes. (TIF) [file pgen.1009958.s006.tif]
