# Supplementary material for: Cell competition is driven by Xrp1-mediated phosphorylation of eukaryotic initiation factor 2α
Source: PLoS Genet. 2021 Dec 6;17(12):e1009958. doi: 10.1371/journal.pgen.1009958 (PMC8675920; doi:10.1371/journal.pgen.1009958)
Supplement: S1 Text — (DOCX) [file pgen.1009958.s007.docx]

**Detailed genotypes used in each figure**

**Fig 1**

(A) eyFLP1 / + or Y; Ubi-GFP, FRT40A / FRT40A

(B) eyFLP1 / + or Y; Ubi-GFP, *wol ^ccp-28^*, FRT40A / FRT40A

(C) GMR-hid, FRT40A, *l (2) CL-L’*/ *wol ^ccp-28^*, FRT40A; ey-Gal4, UAS-FLP

(D) Tub-Gal80, FRT40A / FRT40A; eyFLP6, Act>y+>Gal4, UAS-GFP /+

(E) Tub-Gal80, FRT40A / *wol ^ccp-28^*, FRT40A; eyFLP6, Act>y+>Gal4, UAS-GFP /+

(F) Tub-Gal80, FRT40A / *wol ^ccp-28^*, FRT40A; eyFLP6, Act>y+>Gal4, UAS-GFP / UAS-p35

(G) Tub-Gal80, FRT40A / *wol ^ccp-28^*, FRT40A; eyFLP6, Act>y+>Gal4, UAS-GFP / UAS-wol

(I, J, J’) UbxFlp; Tub-Gal80, FRT40A / *wol ^ccp-28^*, FRT40A; Act>y+>Gal4, UAS-GFP /+

(L-L”) Tub-Gal80, FRT40A / FRT40A; UAS-His2AmRFP, eyFLP6, Act>y+>Gal4 / UAS-Xbp1-GFP

(M-M”) Tub-Gal80, FRT40A / *wol ^ccp-28^*, FRT40A; UAS-His2AmRFP, eyFLP6, Act>y+>Gal4 /UAS-Xbp1-GFP

**Fig 2**

(A-A”) Tub-Gal80, FRT40A / *wol ^ccp-28^*, FRT40A; eyFLP6, Act>y+>Gal4, UAS-GFP /+

(B-B”) eyFLP1, UAS-Dicer2 / + or Y; Tub-Gal80, FRT40A / *wol ^ccp-28^*, FRT40A; Act>y+>Gal4, UAS-GFP / UAS-PERK-RNAi

(E-E”) Tub-Gal80, FRT40A / *wol ^ccp-2^*^8^, FRT40A; eyFLP6, Act>y+>Gal4, UAS-GFP /+

(F-F”) eyFLP1, UAS-Dicer2 / + or Y; Tub-Gal80, FRT40A / *wol ^ccp-28^*, FRT40A; Act>y+>Gal4, UAS-GFP / UAS-PERK-RNAi

**Fig 3**

(A-A”) Tub-Gal80, FRT40A / *wol ^ccp-28^*, FRT40A; eyFLP6, Act>y+>Gal4, UAS-GFP / Xrp1-lacZ

(B-B” and E-E”) eyFLP1, UAS-Dicer2 / + or Y; Tub-Gal80, FRT40A / *wol ^ccp-28^*, FRT40A; Act>y+>Gal4, UAS-GFP / UAS-Xrp1-RNAi

(F-F”) eyFLP5, Act>y+>Gal4, UAS-GFP / UAS-Xrp1^short^; 82B / 82B

(G-G”) eyFLP5, Act>y+>Gal4, UAS-GFP / UAS-Xrp1^short^; 82B / UAS-PERK-RNAi, 82B

**Fig 4**

(A) hs-FLP, UAS-GFP::CD8 / + or Y ;; *M^RpL14^*/+, salE>gRpL14>Gal4 / +

(B) hs-FLP, UAS-GFP::CD8 / + or Y ;; *M^RpL14^*/ UAS-Dicer2, salE>gRpL14>Gal4 / UAS-Xrp1-RNAi

(C) hs-FLP, UAS-GFP::CD8 / + or Y ;; *M^RpL14^*/ UAS-Dicer2, salE>gRpL14>Gal4 / UAS-PERK-RNAi

(E) Tub-Gal80, FRT40A / *Hel25E ^ccp-8^*, FRT40A; eyFLP6, Act>y+>Gal4, UAS–GFP /+

(F) eyFLP1, UAS-Dicer2 / + or Y; Tub-Gal80, FRT40A / *Hel25E ^ccp-8^*, FRT40A; Act>y+>Gal4, UAS-GFP / UAS-Xrp1-RNAi

(G) eyFLP1, UAS-Dicer2 / + or Y; Tub-Gal80, FRT40A / *Hel25E ^ccp-8^*, FRT40A; Act>y+>Gal4, UAS-GFP / UAS-PERK-RNAi

**Fig 5**

(A-A”) hs-FLP, UAS-GFP::CD8 / + or Y ;; *M^RpL14^*/+, salE>gRpL14>Gal4 / Xrp1-lacZ

(B-B”) hs-FLP, UAS-GFP::CD8 / + or Y ;; *M^RpL14^*/+, salE>gRpL14>Gal4 / +

(C-C”) Tub-Gal80, FRT40A / *Hel25E ^ccp-8^*, FRT40A; eyFLP6, Act>y+>Gal4, UAS–GFP / Xrp1-lacZ

(D-D”) Tub-Gal80, FRT40A / *Hel25E ^ccp-8^*, FRT40A; eyFLP6, Act>y+>Gal4, UAS–GFP /+

(I-I”) hs-FLP, UAS-GFP::CD8 / + or Y ;; *M^RpL14^*/ UAS-Dicer2, salE>gRpL14>Gal4 / UAS-PERK-RNAi

(J-J”) hs-FLP, UAS-GFP::CD8 / + or Y ;; *M^RpL14^*/ UAS-Dicer2, salE>gRpL14>Gal4 / UAS-Xrp1-RNAi

(K-K”) eyFLP1, UAS-Dicer2 / + or Y; Tub-Gal80, FRT40A / *Hel25E ^ccp-8^*, FRT40A; Act>y+>Gal4, UAS-GFP / UAS-PERK-RNAi

(L-L”) eyFLP1, UAS-Dicer2 / + or Y; Tub-Gal80, FRT40A / *Hel25E ^ccp-8^*, FRT40A; Act>y+>Gal4, UAS-GFP / UAS-Xrp1-RNAi

**S1 Fig**

(B-B”) Tub-Gal80, FRT40A / *wol ^ccp-28^*, FRT40A; eyFLP6, Act>y+>Gal4, UAS-GFP / UAS-PARP-Vinus

(C) (left panel) eyFLP1 / + or Y; Ubi-GFP, *Elp ^ccp-2^*, FRT40A / FRT40A

(middle panel) GMR-hid, FRT40A, l (2) CL-L’/ *Elp ^ccp-^*^2^, FRT40A; ey-Gal4, UAS-FLP

(right panel) Tub-Gal80, FRT40A / *Elp ^ccp-2^*, FRT40A; UAS-His2AmRFP, eyFLP6, Act>y+>Gal4 /UAS-Xbp1-GFP

(D) (left panel) eyFLP1 / + or Y; Ubi-GFP, *Calr ^ccp-21^*, FRT40A / FRT40A

(middle panel) GMR-hid, FRT40A, l (2) CL-L’/ *Calr ^ccp-21^*, FRT40A; ey-Gal4, UAS-FLP

(right panel) Tub-Gal80, FRT40A / *Calr ^ccp-21^*, FRT40A; UAS-His2AmRFP, eyFLP6, Act>y+>Gal4 /UAS-Xbp1-GFP

(E) (left panel) GMR-hid, FRT40A, *l (2) CL-L’*/ FRT40A; ey-Gal4, UAS-FLP/UAS-Xbp1-GFP

(right panel) GMR-hid, FRT40A, *l (2) CL-L’*/ *wol ^ccp-28^*, FRT40A; ey-Gal4, UAS-FLP/UAS-Xbp1-GFP

(F) (left panel) GMR-hid, FRT40A, *l (2) CL-L’*/ FRT40A; ey-Gal4, UAS-FLP

(middle panel) GMR-hid, FRT40A, *l (2) CL-L’*/ *wol ^ccp-28^*, FRT40A; ey-Gal4, UAS-FLP

**S2 Fig**

(A) eyFLP1, UAS-Dicer2 / + or Y; Tub-Gal80, FRT40A / *wol ^ccp-28^*, FRT40A; Act>y+>Gal4, UAS-GFP / UAS-Gcn2-RNAi

**S3 Fig**

(A-A”) Tub-Gal80, FRT40A / *wol ^ccp-28^*, FRT40A; UAS-His2AmRFP, eyFLP6, Act>y+>Gal4 /UAS-Xbp1-GFP, UAS-Xrp1-RNAi

**S4 Fig**

(A)Tub-Gal80, FRT40A / FRT40A; eyFLP6, Act>y+>Gal4, UAS-GFP /+

(B) eyFLP1, UAS-Dicer2 / + or Y; Tub-Gal80, FRT40A / FRT40A; Act>y+>Gal4, UAS-GFP / UAS-PERK-RNAi

(C) nub-gal4, UAS-GFP; UAS-Xrp1^short^ / +

(D) nub-gal4, UAS-GFP / UAS-p35; UAS-Xrp1^FlyORF^/ UAS-PERK-RNAi

**S5 Fig**

(A) hs-FLP, UAS-GFP::CD8 / + or Y ;; *M^RpL14^*/ UAS-Dicer2, salE>gRpL14>Gal4 / UAS-PERK-RNAi, Xrp1-lacZ

(B) eyFLP1, UAS-Dicer2 / + or Y; Tub-Gal80, FRT40A / *Hel25E ^ccp-8^*, FRT40A; Xrp1-lacZ, Act>y+>Gal4, UAS-GFP / UAS-PERK-RNAi

**S6 Fig**

(A) nub-gal4, UAS-GFP; Xrp1-lacZ/+

(B) nub-gal4, UAS-GFP; Xrp1-lacZ/Tub-Gal80[ts], UAS-PERK
