# Supplementary material for: Cell competition is driven by Xrp1-mediated phosphorylation of eukaryotic initiation factor 2α
Source: PLoS Genet. 2021 Dec 6;17(12):e1009958. doi: 10.1371/journal.pgen.1009958 (PMC8675920; doi:10.1371/journal.pgen.1009958)
Supplement: Attachment — Submitted filename: Response to Reviewers.ver2.pdf [file pgen.1009958.s008.pdf]

Responses to Reviewers' comments

We would like to thank the reviewers for excellent comments, which have led us to improve our manuscript substantially. Modifications are indicated in red in the revised manuscript.

Reviewer #1:

Summary

In this manuscript, the authors show that mutations leading to ER stress are able to induce cell competition, which confirms previous studies that proteotoxic stresses is a driving force in Minute based cell competition (Baumgartner et al.,2021). In an EMS screen for cell competition factors they found genes involved in ER stress and the UPR. They then argue that these factors induce cell competition. Building on the connection they position Xrp1 upstream of the stress response. Increased expression of Xrp1 is suggested to increase of peIF2α levels, which in turn reduce protein translation.

The novelty of this paper derives from the description of the link between Xrp1 and PERK/ peIF2α. This connection will be of general interest. The report will certainly stimulate further work. Unfortunately, not all the conclusions are equally well substantiated. Additional experiments and/or extended discussion would be beneficial.

Major comments:

- The title of the paper is "Cell competition is driven by Xrp1-mediated phosphorylation of eukaryotic initiation factor 2α". The implied "Xrp1-PERK-eIF2α axis" places Xrp1 upstream of PERK. While intriguing the provided evidence does not exclude that Xrp1 acts in a positive feedback loop. Based on the timing of the experiments can the authors exclude this alternative? How do the authors envisage that Xrp1 mediates the phosphorylation of eukaryotic initiation factor 2α? Xrp1 is a nuclear/nucleolar transcription factor PERK located on the ER membrane. Additional experiments and discussion should be provided or the title adjusted.

Response:

We thank the reviewer for the comment. As pointed out by the reviewer, our data do not exclude the possibility that Xrp1 also acts downstream of PERK in a positive feedback loop. Indeed, we found that overexpression of PERK in the wing disc led to upregulation of Xrp1 expression (please see new Fig. S6). In addition, recent studies have shown that

overexpression of PERK or ATF4, a transcription factor the translation of which is induced by phosphorylated eIF2α, causes upregulation of Xrp1 (Langton *et al.*, bioRxiv, 2021; Brown *et al.*, *eLife*, 2021). These observations suggest that Xrp1 can act both upstream and downstream of the PERK-eIF2α axis in a positive feedback loop. However, we also found that induction of *Xrp1* expression in *RpL14/+* or *Hel25E*$^{-/-}$ loser clones was not suppressed by PERK knockdown (please see new Fig. S5, quantified in new Fig. 5E and G), indicating that Xrp1 mainly acts upstream of PERK in these loser clones.

As for the comment “How do the authors envisage that Xrp1 mediates the phosphorylation of eukaryotic initiation factor 2α?”, it is possible that expression of PERK is upregulated by Xrp1, as shown by Kiparaki et al. (bioRxiv, 2021).

We have now included these new data and discussions in the revised manuscript as follows:

(page 9, Discussion)
“Notably, Xrp1 has been implicated to be a functional homolog of mammalian CHOP, a transcription factor that is induced by ATF4 [29]. Consistently, we found that overexpression of PERK leads to upregulation of Xrp1 expression (Fig. S6). In addition, recent studies have shown that overexpression of PERK or ATF4 upregulates Xrp1 [32, 34]. These observations suggest that Xrp1 acts both upstream and downstream of the PERK-eIF2α axis in a positive feedback loop. The upstream Xrp1 may activate the PERK-eIF2α axis via upregulation of PERK expression [33]. Alternatively, Xrp1 upregulation may cause ER stress, which induces PERK activation. These are also important issues that should be addressed in the future studies.”

(page 8)
“The induction of *Xrp1* expression in loser clones was not suppressed by PERK knockdown (Fig. S5A and B, quantified in Fig. 5E and G), indicating that Xrp1 acts upstream of PERK in these clones.”

- The key experiments for their claim are presented in Figure 3. Xrp1 knock-down and overexpression are presented. In a wol-/- clone p-eIF2α levels are increased. This is reduced when Xrp1 is knocked down. Overexpression of Xrp1 increases p-eIF2α levels.

The presented results are suggestive, but the quantification suggests a high degree of variability. An increased sample size would be helpful. Alternatively, it could help to express Xrp1 in, for example, the posterior compartment and compare the resulting p-eIF2α levels to those in the anterior compartment. There seems to be a significant variation in the background levels of p-eIF2α and this alternative approach may simplify the analysis and interpretation.

Response:

We thank the reviewer for the comment. Following the reviewer's suggestion, we have now increased the sample size for the p-eIF2α immunostaining analysis (please see new Fig. 3D, below; the sample size was increased from n=13 to n=26). We believe that the data are now very clear and support our conclusion.

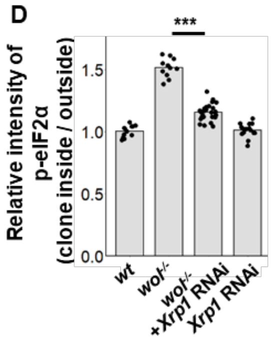

Fig. 3D

(D) Quantification of the size of GFP-labeled clones shown in Fig.3B. Error bars, SD; ***$p<0.001$ by Welch's t-test.

In addition, we have now provided new data that overexpression of Xrp1 in the wing pouch results in elevation of p-eIF2α levels compared to other parts of the wing disc (Fig. S4, shown below).

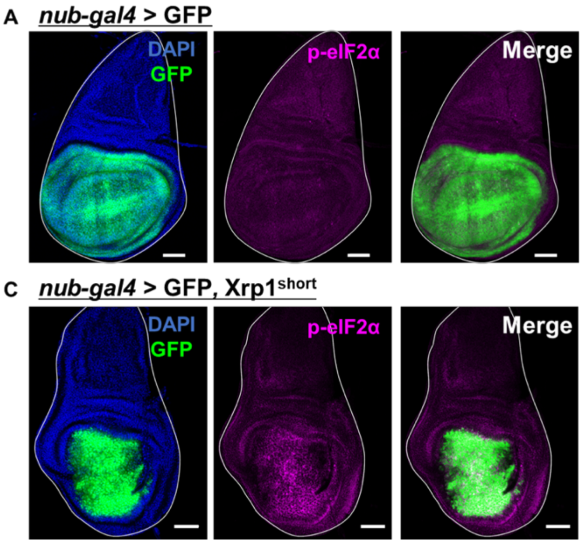

Fig. R1A (only for the reviewer) and Fig. S4C
(A) Wing disc overexpressing GFP in the wing pouch by the *nub-Gal4* driver stained with anti-phosphorylated eIF2α.
(C) Wing disc overexpressing GFP, $Xrp1^{short}$ in the wing pouch by the *nub-Gal4* driver stained with anti-phosphorylated eIF2α.

We have now included these new data in the revised manuscript as follows:

(page 7)
"Moreover, overexpression of Xrp1 was sufficient to induce phosphorylation of eIF2α (Fig. 3F, compare to Fig. S4A, quantified in Fig. 3H; Fig. S4C)."

Also, not all wol -/- clones seem to have elevated p-eIF2α (Figure 2a). This should be discussed.

Response:
As indicated by the reviewer, most, but not all, of $wol^{-/-}$ clones elevate p-eIF2α staining (Fig. 2A). This could be due to the fact that the extent of ER stress (Xbp1-GFP) also varied among clones (Fig. 1M). Accordingly, we have now modified the text in the revised manuscript as follows:

(page 6)
"As expected, immunostaining analysis showed that most *wol* mutant clones elevate the phosphorylation of eIF2α (Fig. 2A, quantified in Fig. 2C), an indication of UPR activation [25]."

- The data in figure 3F-H is not convincing, because only a weak upregulation of p-eIF2α can be seen upon Xrp1 overexpression. To exclude trivial explanations the authors should compare p-eIF2α expression in a control with a random UAS line (e.g. GFP or LacZ) and also the effect of PERKRNAi without Xrp1 overexpression. As Xrp1 overexpressing clones are very small, they could either induce bigger clones or show the effect of Xrp1 overexpression in compartments.

Response:
We thank the reviewer for the comment. Following the reviewer's suggestion, we have now added control data for the p-eIF2α immunostaining of the discs bearing clones expressing GFP alone or PERK-RNAi without Xrp1. As shown in new Fig. S4 (please

see below), GFP expression alone did not affect the p-eIF2a levels and expression of PERK-RNAi downregulated the endogenous level of p-eIF2a. We have also provided the p-eIF2α immunostaining data of the wing disc overexpressing Xrp1 in the wing pouch (Fig. S4C, as described above).

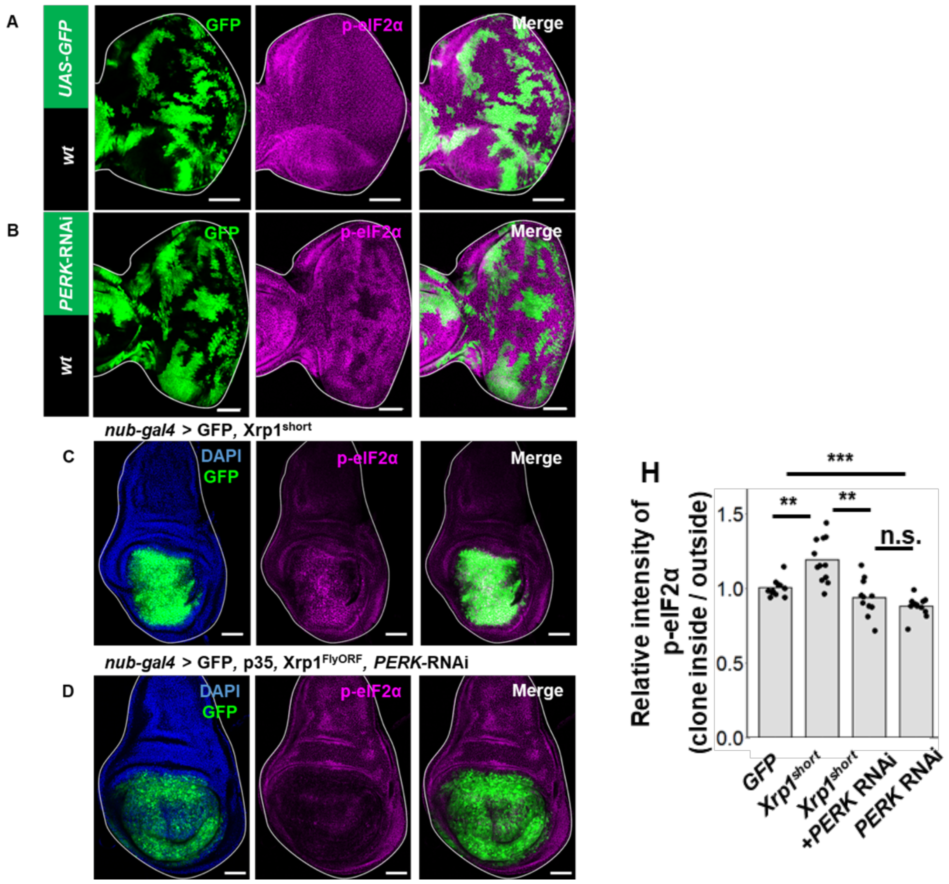

Fig. S4 and Fig. 3H

(A) Eye disc bearing eyFLP-induced MARCM clones of UAS-GFP cells stained with anti-phosphorylated eIF2α.

(B) Eye disc bearing eyFLP-induced MARCM clones of PERK RNAi cells stained with anti-phosphorylated eIF2α.

(C) Wing disc overexpressing GFP, Xrp1^{short} in the wing pouch by the *nub-Gal4* driver stained with anti-phosphorylated eIF2α.

(D) Wing disc overexpressing GFP, Xrp1^{FlyORF} in the wing pouch by the *nub-Gal4* driver stained with anti-phosphorylated eIF2α.

(H) Quantification of the intensity of anti-phosphorylated eIF2α staining in clones shown in F, G and Fig. S4B. Error bars, SD; ***$p<0.001$ by Steel-Dwass test.

Scale bars, 50µm.

We have now included these new data in the revised manuscript as follows:

(page 7)

"Moreover, overexpression of Xrp1 was sufficient to induce phosphorylation of eIF2α (Fig. 3F, compare to Fig. S4A, quantified in Fig. 3H; Fig. S4C). Furthermore, Xrp1-induced phosphorylation of eIF2α was canceled by PERK knockdown (Fig. 3G, compare to Fig. S4B, quantified in Fig. 3H; Fig. S4D)."

- They use two different UAS-lines of Xrp1 (Figure 3 and Figure S3), in which they name Xrp1 differently (Xrp1short and Xrp1). They should clarify the difference between the two lines and prove that both lines lead to the same phenotype.

Response:

We thank the reviewer for the comment. There are two isoforms of Xrp1, namely $Xrp1^{long}$ and $Xrp1^{short}$, overexpression of which show the same phenotype (Boulan *et al., Dev Cell*, 2019). The UAS-$Xrp1^{short}$ transgene used in this study produces the $Xrp1^{short}$ isoform (Boulan *et al., Dev Cell*, 2019). The UAS-$Xrp1^{FlyORF}$ transgene (FlyORF: F000655) produces even shorter form of the Xrp1 protein than the $Xrp1^{short}$ isoform; however, as shown below (Fig. R2 for the reviewer), we found that both UAS-$Xrp1^{short}$ and UAS-$Xrp1^{FlyORF}$ fly lines showed exactly the same results in the p-eIF2α immunostaining experiments.

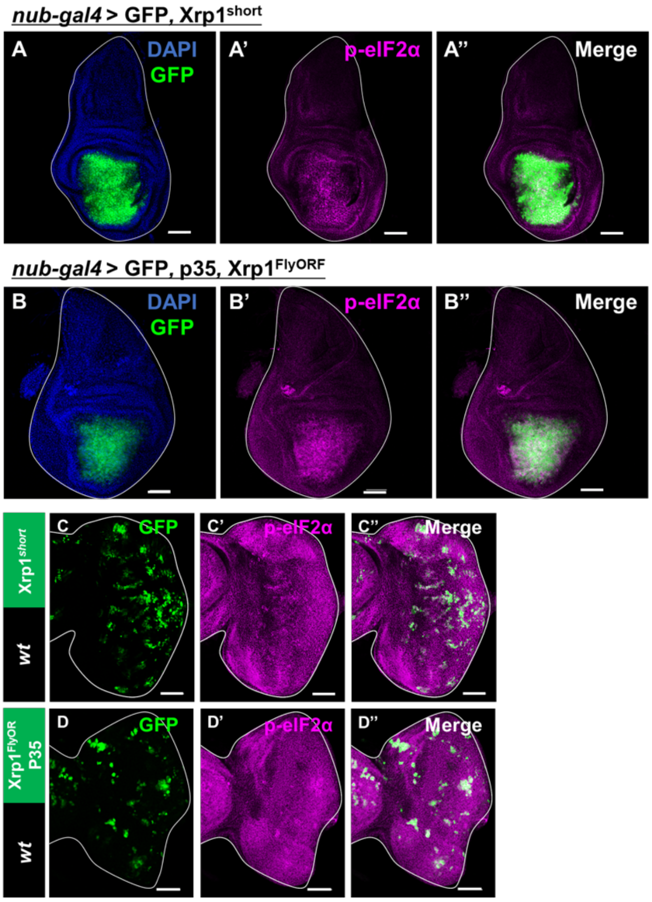

Fig. R2
(A-A") Wing disc overexpressing GFP and Xrp1^{short} in the wing pouch by the *nub-Gal4* driver stained with anti-phosphorylated eIF2α.
(B-B") Wing disc overexpressing GFP and Xrp1 in the wing pouch by the *nub-Gal4* driver stained with anti-phosphorylated eIF2α.
(C-C") Eye disc bearing eyFLP-induced MARCM clones of UAS-Xrp1^{short} cells stained with anti-phosphorylated eIF2α.
(D-D") Eye disc bearing eyFLP-induced MARCM clones of UAS-Xrp1^{FlyORF} + p35 cells stained with anti-phosphorylated eIF2α.
Scale bars, 50μm.

We have now presented the data using the UAS-Xrp1^{short} transgene in the main figure (Fig. 3) and cited a reference for it as follows:

(page 14, Figure 3 legend)
"(F and G) Eye disc bearing eyFLP-induced MARCM clones of UAS-Xrp1^{short} (F) [46] or UAS-Xrp1^{short} + PERK-RNAi (G) cells stained with anti-phosphorylated eIF2α."

- Data shown in figure 5 is not convincing. Their depicted Minute clone in 5a does not reflect the data of their graph in 5e. In Figure 5, they should show full discs containing several clones for all experiments. Showing additional close-ups of clones would also help. To corroborate the main message of the paper: the authors should quantify how the Xrp1 LacZ and peIF2α intensities are changing during cell competition when using Xrp1RNAi or PERKRNAi.

<u>Response:</u>
We thank the reviewer for the comment. Following the reviewer's suggestion, we have now shown the full discs for all the experiments in Figure 5 (please see new Fig. 5, below). In addition, we have now provided the quantitative data for Xrp1-LacZ and p-eIF2α staining when Xrp1-RNAi or PERK-RNAi is expressed (please see new Fig. 5 and Fig. S5, shown below). We have now included these new data in the revised manuscript as follows:

(page 8)
"The induction of *Xrp1* expression in loser clones was not suppressed by PERK knockdown (Fig. S5A and B, quantified in Fig. 5E and G), indicating that Xrp1 acts

upstream of PERK in these clones. On the other hand, the elevation of eIF2α phosphorylation was abolished when *PERK* or *Xrp1* was knocked down in these loser clones (Fig. 5I-L, quantified in Fig. 5F and H).”

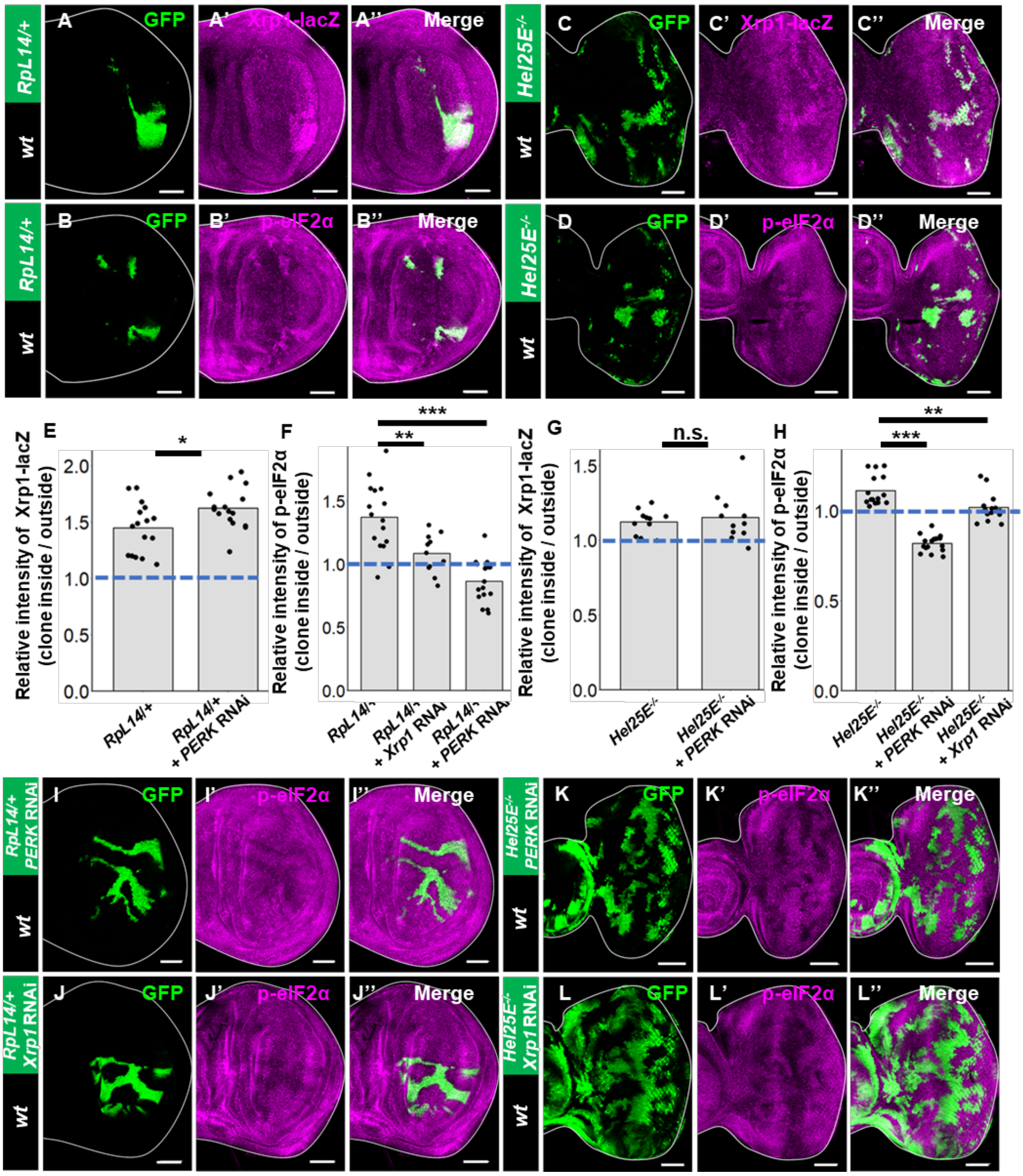

Fig. 5

(A-A”) Xrp1-lacZ/+ background wing disc bearing hsFLP-induced GFP-labeled clones of RpL14/+, salE>GFP cells stained with anti-β-gal.

(B-B”) Wild-type background wing disc bearing hsFLP-induced GFP-labeled clones of RpL14/+, salE>GFP cells stained with anti-phosphorylated eIF2α.

(C-C”) Xrp1-lacZ/+ background eye disc bearing eyFLP-induced MARCM clones of *Hel25E*$^{-/-}$ cells stained with anti-β-gal.
(D-D”) Wild-type background eye disc bearing eyFLP-induced MARCM clones of *Hel25E*$^{-/-}$ cells stained with anti-phosphorylated eIF2α.
(E) Quantification of the intensity of anti-β-gal staining in clones shown in A and S5.A. Error bars, SD; ***p<0.001 by Welch’s t-test.
(F) Quantification of the intensity of anti-phosphorylated eIF2α staining in clones shown in B-D. Error bars, SD; ***p<0.001 by Dunnett test.
(G) Quantification of the intensity of anti-β-gal staining in clones shown in E and S5.B. Error bars, SD; ***p<0.001 by Dunnett test.
(H) Quantification of the intensity of anti-phosphorylated eIF2α staining in clones shown in F-H. Error bars, SD; ***p<0.001 by Dunnett test.
Scale bars, 50µm.
(I-I”) Wild-type background wing disc bearing hsFLP-induced GFP-labeled clones of RpL14/+, salE>GFP + PERK RNAi cells stained with anti-phosphorylated eIF2α.
(J-J”) Wild-type background wing disc bearing hsFLP-induced GFP-labeled clones of RpL14/+, salE>GFP + Xrp1 RNAi cells stained with anti-phosphorylated eIF2α.
(K-K”) Wild-type background eye disc bearing eyFLP-induced MARCM clones of *Hel25E*$^{-/-}$ + PERK RNAi cells stained with anti-phosphorylated eIF2α.
(L-L”) Wild-type background eye disc bearing eyFLP-induced MARCM clones of *Hel25E*$^{-/-}$ + Xrp1 RNAi cells stained with anti-phosphorylated eIF2α.

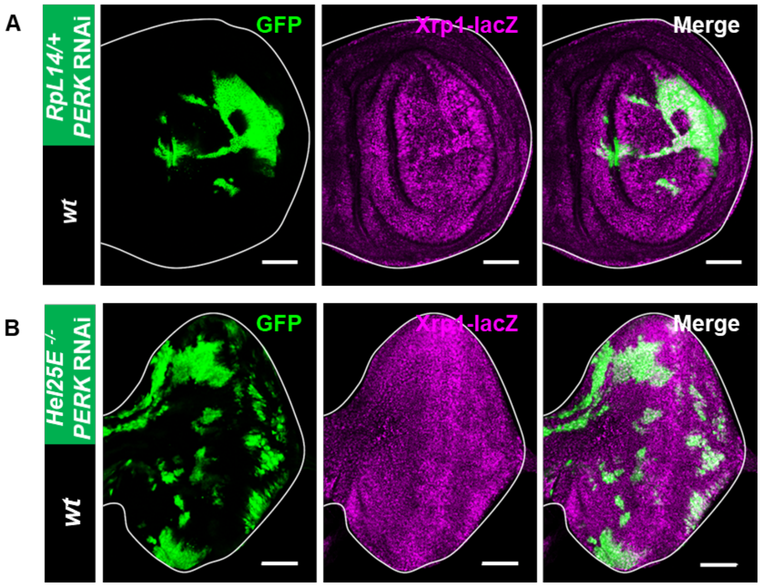

Fig. S5
(A) Xrp1-lacZ/+ background wing disc bearing hsFLP-induced GFP-labeled clones of RpL14/+, salE>GFP + PERK-RNAi cells stained with anti-β-gal.
(B) Xrp1-lacZ/+ background eye disc bearing eyFLP-induced MARCM clones of *Hel25E*$^{-/-}$ + PERK RNAi cells stained with anti-β-gal.
Scale bars, 50µm.

Minor comments:

- In places the text seems hastily assembled, which detracts unnecessarily from the otherwise thought-provoking data. The authors should carefully revise the text, as it is not updated on recent literature from their field and their citation style is not consistent. At the beginning of this year two studies have independently shown that proteotoxic stress is induced by a Minute situation and is essential for cell competition (Baumgartner et al., 2021, Recasens-Alvarez et al., 2021). Citing these is needed to properly place the claims in the context of the current literature.

Response:
We thank the reviewer for the comment. We have now cited these studies and discussed them in the Discussion section in the revised manuscript as follows:

(page 8)
"Importantly, our data show that eIF2α phosphorylation is also required for the induction of loser's death. Similarly, recent studies have shown that *M*/+ cells experience proteotoxic stress and thus induce phosphorylation of eIF2α, which acts as a driver of *M*/+ cell competition [30-33]. Whether the global inhibition of protein synthesis or other downstream event(s) of eIF2α phosphorylation such as upregulation of UPR-activating transcription factor ATF4 is linked to their apoptosis is an outstanding important question."

- Authors should try to explain the link of Xrp1 and PERK in more detail. They should try to integrate what is known of endoplasmic reticulum unfolded protein responses and how this leads to an increased peIF2α. Which model would the authors propose how Xrp1 functions in their PERK-Xrp1- peIF2α model? What factors could be upstream of Xrp1? Blanco et al., proposed that the mammalian homolog of Xrp1 might be CHOP/ddit3. This would put Xrp1 below PERK. Discussing this would help place the claims in context.

Response:
We thank the reviewer for the comment. As for the former comment "They should try to integrate what is known of endoplasmic reticulum unfolded protein responses and how this leads to an increased peIF2α", we have now included this information in the

Introduction section in the revised manuscript as follows:

(page 4)
"The stresses are sensed by four specialized kinases (PERK, GCN2, PKR, and HRI) that converge on phosphorylation of the alpha subunit of the eukaryotic translation initiation factor 2 (eIF2α). For instance, upon accumulation of unfolded proteins in the ER, the ER-resident chaperone BiP/Hsc70-3 is released from PERK, leading to homodimerization and activation of the eIF2α kinase PERK."

As for the latter comment, a possible mechanism by which Xrp1 functions upstream of PERK is that Xrp1 upregulates PERK transcription directly or indirectly, as recently shown by Nick Baker's lab (Kiparaki et al., bioRxiv, 2021). Alternatively, it is also possible that upregulation of Xrp1 causes ER stress, which induces PERK activation. Indeed, we found that overexpression of Xrp1 resulted in the induction of ER stress as visualized by the Xbp1-GFP marker (please see Fig. R3, below). Although it is possible that this induction of ER stress is due to an overexpression effect, these data support our hypothesis.

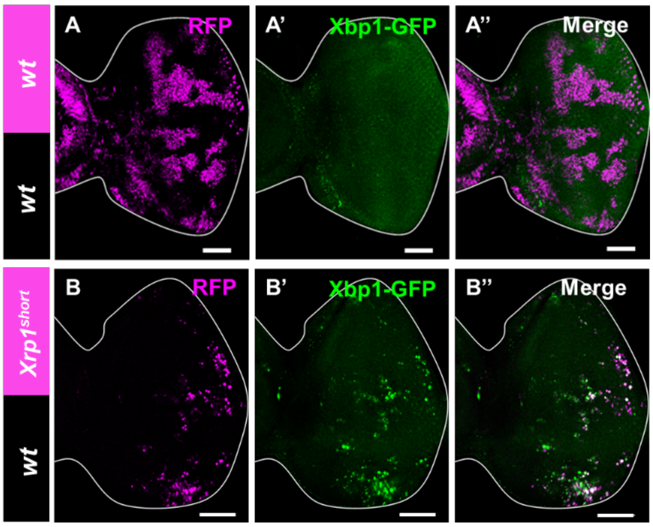

Fig. R3
(A-A") Eye disc bearing eyFLP-induced MARCM clones of UAS-Xbp1-GFP cells stained with anti-GFP.
(B-B") Eye disc bearing eyFLP-induced MARCM clones of UAS-Xrp1^{short}+UAS-Xbp1-GFP cells stained with anti-GFP.
Scale bars, 50µm.

We have now included these discussions in the revised manuscript as follows:

(page 9)

“Notably, Xrp1 has been implicated to be a functional homolog of mammalian CHOP, a transcription factor that is induced by ATF4 [29]. Consistently, we found that overexpression of PERK leads to upregulation of Xrp1 expression (Fig. S6). In addition, recent studies have shown that overexpression of PERK or ATF4 upregulates Xrp1 [32, 34]. These observations suggest that Xrp1 acts both upstream and downstream of the PERK-eIF2α axis in a positive feedback loop. The upstream Xrp1 may activate the PERK-eIF2α axis via upregulation of PERK expression [33]. Alternatively, Xrp1 upregulation may cause ER stress, which induces PERK activation. These are also important issues that should be addressed in the future studies.”

- A further missing reference connecting ER stress and Xrp1 is Mallik et al.,2018. They reported a role for Xrp1 in the toxicity of the ALS-associated FUS orthologue caz mutant phenotype.

Response:

We thank the reviewer for the comment. We have now described the role of Xrp1 in the toxicity of the ALS-associated FUS orthologue caz mutant and discussed the connection between ER stress, Xrp1, and cell competition in the revised manuscript as follows:

(page 9)

“Thus, our finding that the Xrp1-PERK-eIF2α axis commonly drives cell competition has opened the way to understanding the physiological and pathological role of cell competition. Intriguingly, mutations in the fused in sarcoma (FUS) gene, which are linked to amyotrophic lateral sclerosis (ALS), cause ER stress [40] and the Drosophila FUS orthologue cabeza genetically interacts with Xrp1[41]. In addition, it has been shown that cell competition plays a role in neurodegenerative diseases [44, 45], which are thought to be driven by ER stress.”

- Given the narrowness of the clones in Figure 1J/K, it is somewhat debatable to discuss apoptosis on the border vs middle of the clone. Hopefully alternative images could be provided.

<u>Response:</u>
We thank the reviewer for the comment. As pointed out by the reviewer, clones are small and thus it is not very easy to distinguish apoptosis on the border vs. middle of the clones at low magnification. However, by analyzing higher magnification images, we could distinguish them (quantified in Fig. 1K). Although we reproducibly obtained similar data shown in Fig. 1J (which is an average image), we would like to emphasize that *wol*$^{-/-}$ clones with anti-cDcp1 staining are very different form clones that cause autonomous apoptosis such as reaper-overexpressing clones. We also would like to show another example of *wol*$^{-/-}$ clones stained with anti-cDcp1 antibody for the reviewer below (please see Fig. R4).

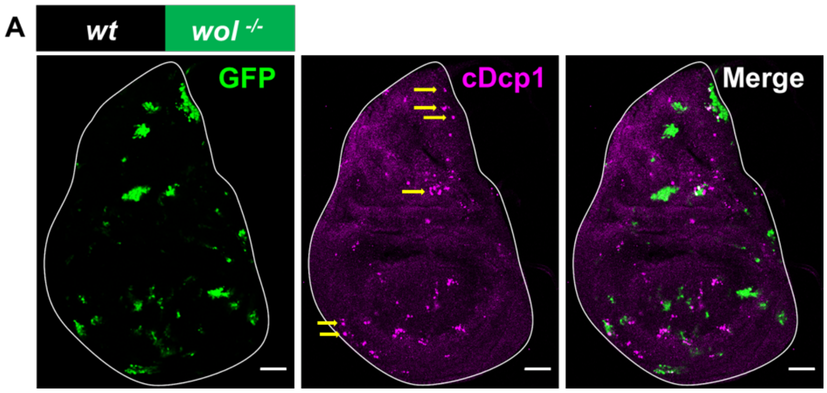

Fig. R4
(A) Wing disc bearing UbxFLP-induced MARCM clones of *wol*$^{-/-}$ cells stained with anti-cleaved Dcp-1. Arrows indicate typical examples of dying cells at the clone boundary. Scale bars, 50µm.

<u>Reviewer #2:</u>
Ochi, Igaki review:

In this very nice body of work, the authors carried out an EMS-based genetic screen using mitotic clones in the developing Drosophila eye to look for inducers of cell competition. They screened 12,500 mutant chromosomes and identified 87 mutations that caused cells to be competitively eliminated from mosaic eye (named ccp mutants). Genomic

sequencing revealed mutations in three genes involved in ER stress: Elp3, calreticulin, and wollknaeuel (wol), involved in N-linked protein glycosylation in ER. Focusing on wol mutants, they found that mutants did not alter normal eye size (but see comments 1 and 2 below), but when with WT cells in mosaic eye discs wol mutant cells were eliminated via apoptosis (p35 prevented their elimination). Cell death was observed at clone boundaries, which is also seen in many contexts of cell competition.

The authors found that Xrp1 was upregulated in the wol clones, and p-eIF2a was increased, consistent with activation of the integrated stress response; accordingly, a reduction in OPP signal in the clones indicated that protein synthesis was downregulated. Clonal Xrp1 over-expression led to similar increases in p-eIF2a, which was suppressed by co-expression of PERK-RNAi. All of these results are consistent with activation of the ISR due to ER stress in the mutant cells. The authors then asked if ISR activation occurred in other competitive contexts by looking at mosaic discs containing clones of either Rpl14+/- cells, or Hel25E-/- cells. The mutant cells of both genotype are normally out competed by the WT cells, but when either Xrp1 or the ER stress kinase PERK was knocked down in the mutant clones, the cells were no longer eliminated. Altogether, their data suggest that the Xrp1-PERK-eIF2α axis is commonly activated in these different genetic contexts of cell competition, leading to a reduction in protein synthesis and induction of apoptosis in the loser cells. Intriguingly, GCN2, another ER stress sensing eIF2a kinase, was found not to be required, suggesting some interesting specificity.

The experiments in the paper are well done and nicely presented. The paper is also well written and for the most part the authors are appropriately circumspect in their conclusions (see comment #4 below). Work published very recently by others showed that Rp haploinsufficiency results in severe proteotoxic stress, activation of the ISR, and increased cell autonomous cell death as well as heightened cell competition in mosaics, and that Xrp1 and its partner Irbp18 are involved (Baumgartner 2021 and Recausins 2021). The work of Igaki and his colleagues work adds to this and represents an important advance to the field, as it describes additional mutations that, along with the Rp+/- group, appear to form a common mechanism underlying cell competition in mosaic tissues.

Comments:

1. Although the very recently published papers from the Piddini and Vincent labs (Baumgartner 2021 and Recausins 2021) are not cited here (but should be), given those lab's results that Rp+/- cells exhibit severe proteotoxic stress even in the absence of cell competition yet survive to form an animal after a developmental delay, I wonder if wol mutants show similar phenotypes: e.g, if the mutant tissues/animals were viable but

developmentally delayed with sporadic cell autonomous cell death in the absence of cell competition. It would be enlightening to look at eye or wing development for timing and for cell death during the larval growth period, if the wol-/- mutants are viable (or if heterozygous wol mutants show increased ER stress).

Response:
We thank the reviewer for the comment. First, we have now cited recently published papers from the Piddini and Vincent labs in the revised manuscript as follows:

(page 8)
"Importantly, our data show that eIF2α phosphorylation is also required for the induction of loser's death. Similarly, recent studies have shown that *M*/+ cells experience proteotoxic stress and thus induce phosphorylation of eIF2α, which acts as a driver of *M*/+ cell competition [30-33]."

The latter comment is very interesting. However, unfortunately, *wol*$^{-/-}$ mutant flies are lethal and thus we cannot analyze cell behaviors or developmental timing of *wol*$^{-/-}$ mutant flies. In addition, heterozygous *wol* mutants do not show increased ER stress; as shown below, MARCM-induced *wol*$^{-/-}$ mosaic tissue shows Xbp1p>dsRed signal (ER stress marker, Ryoo *et al.,* PLOS ONE., 2013) strictly in a clone-autonomous manner (which indicates that ER stress is not induced in *wol*$^{+/-}$ heterozygous cells) (please see Fig. R5).

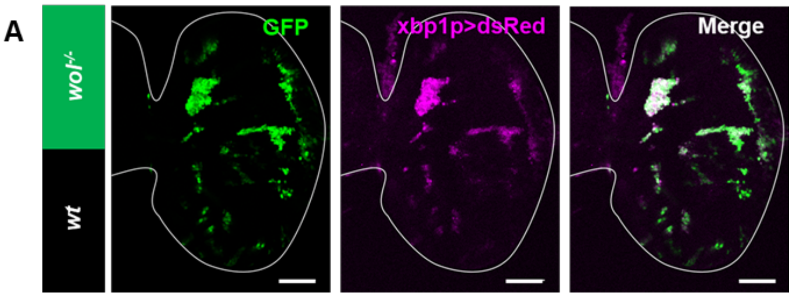

Fig. R5
(A) Xbp1p>dsRed/+ eye disc bearing eyFLP-induced MARCM clones of *wol*$^{ccp\text{-}28\text{-}/\text{-}}$

2. Related to the thoughts in #1, the entirely wol mutant eye shown in Fig. 1 is smaller and looks like it has a reduced number of ommatidia, although they are regularly arrayed. Do the disc cells express Xbp1 or other markers that would indicate that they suffer ER stress? The way the wol mutant eyes were generated for Fig. 1 was via mosaicism coupled

with WT-linked cell-lethality, to eliminate all WT cells from the eye disc. Since the eye seems slightly smaller than normal, could it be that compensatory growth is prevented by the absence of WT cells? If so, is this accompanied by a lack of developmental delay? Either way, such information on cell-autonomous cell death and developmental delay in the mutants would be very informative for the field.

Response:

We thank the reviewer for the thoughtful comment. As for the first comment "Do the disc cells express Xbp1 or other markers that would indicate that they suffer ER stress?", we have now analyzed ER stress in the eye discs entirely mutant for *wol* (with surrounding wild-type tissue removed; Fig. 1C) and found that the Xbp1-GFP signal is indeed broadly upregulated in the *wol*$^{-/-}$ eye discs (please see new Fig. S1E, shown below), indicating that *wol*$^{-/-}$ eye discs suffer ER stress.

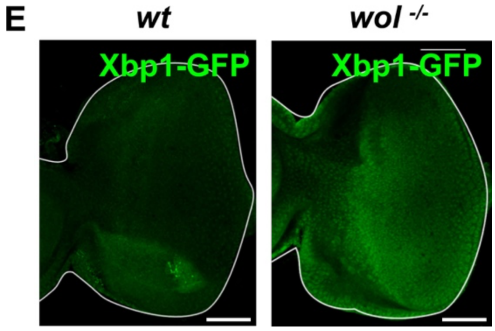

Fig. S1E

(E) Wild-type (left) or *wol*$^{ccp\text{-}28\text{-}/\text{-}}$ (right) eye disc bearing UAS-Xbp1-GFP. In both tissues, wild-type or *wol*$^{ccp\text{-}28\text{-}/\text{-}}$ clones were induced in the eye disc and then surrounding wild-type tissue was removed by GMR-hid and cell-lethal mutations. Scale bars, 50μm.

These data suggest that the slightly smaller size of *wol*$^{-/-}$ eyes could be due to an elevated ER stress in the eye discs. Intriguingly, however, we fund that the number of cell death was not significantly increased in *wol*$^{-/-}$ eye discs compared to wild-type eye discs (please see new Fig. S1F, shown below), suggesting that the smaller *wol*$^{-/-}$ eyes is due to a growth defect caused by ER stress.

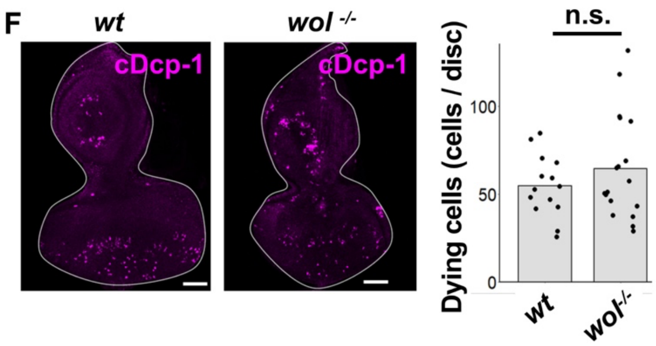

Fig. S1F

Wild-type (left) or *wol*$^{ccp\text{-}28\text{-}/\text{-}}$ (middle) eye disc stained with anti-cleaved Dcp-1. In both tissues, wild-type or *wol*$^{ccp\text{-}28\text{-}/\text{-}}$ clones were induced in the eye disc and then surrounding wild-type tissue was removed by GMR-hid and cell-lethal mutations. Scale bars, 50μm. (right) Quantification of the number of dying cells in wild-type or *wol*$^{ccp\text{-}28\text{-}/\text{-}}$ eye discs. Error bars, SD; ***$p<0.001$ by Welch's t-test.

As for the latter comment "Could it be that compensatory growth is prevented by the absence of WT cells? If so, is this accompanied by a lack of developmental delay?", we have found that cell death was not significantly increased in *wol*$^{-/-}$ eye discs (as shown above) and thus developmental timing would not be affected. Nonetheless, we have analyzed developmental timing (from egg laying to wandering larva) of flies bearing wild-type or *wol*$^{-/-}$ eye discs (with surrounding wild-type tissue removed) and found, intriguingly, that flies with *wol*$^{-/-}$ eye discs show a slight developmental delay (please see Fig. R6, shown below).

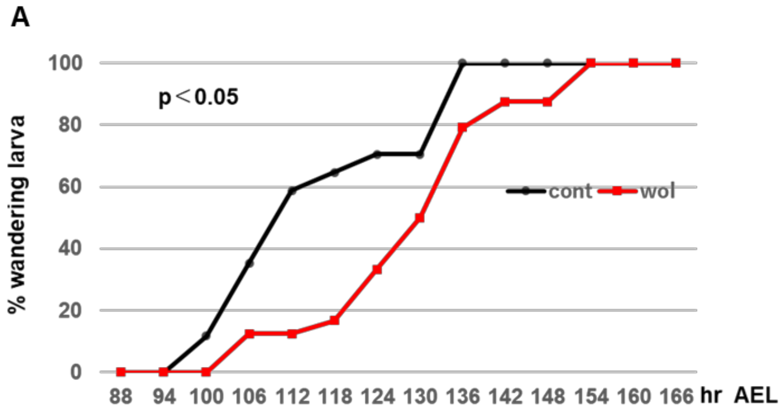

Fig. R6

(A) Developmental timing (from egg laying to wandering larva) of flies with wild-type (n = 17, black line) or *wol*$^{-/-}$ (n = 24, red line) eye discs. AEL; after egg laying, $p<0.05$ (log-rank analysis).

Given that there was no significant increase in the number of dying cells in *wol*$^{-/-}$ eye discs, we do not have a good explanation for this phenomenon at this stage. Thus, we would like to investigate this intriguing phenomenon in the future study.

We have now included these new data and discussions in the revised manuscript as follows:

(page 6)

"The slightly smaller size of *wol*$^{-/-}$ eyes (Fig. 1C) could be due to a broadly increased

ER stress in the entire eye disc (Fig. S1E). Intriguingly, cell death was not significantly increased in the entire *wol*$^{-/-}$ eye discs (Fig. S1F), suggesting that the smaller eye is due to a growth defect caused by elevated ER stress."

3. Are Calr and Elp3 mutants also viable (+/- and/or -/-)?

Response:

Calr$^{-/-}$ and *Elp3*$^{-/-}$ mutant flies are both lethal, unfortunately. Only heterozygous flies are viable for these mutants, and thus we cannot analyze how these loser cells behave when they present in the entire animal.

4. Since not every context of cell competition was tested the title to Supp Fig. 1 seems a bit overstated. A suggesting is to change it to "ER stress underlies several different genetic contexts of cell competition".

Response:

We thank the reviewer for the suggestion. We agree with the reviewer and have now modified the title of Suppl Fig. 1 as shown below in revised manuscript.

(page 15)

"ER stress underlies several different genetic contexts of cell competition"

5. It is possible that the GCN2 RNAi is weak even with the addition of Dicer-2, thus yielding no suppression of competitive elimination of wol mutant cells. Do the authors know how efficient the GCN2 RNAi is in vivo?

Response:

We thank the reviewer for the comment. It has previously been reported that GCN2-RNAi (BDSC #35355, the same allele as we used in this study) reduces the GCN2 mRNA levels (as assessed by RT-PCR) and suppresses the amino acid response pathway *in vivo* (Armstrong *et al*., *Development,* 2014). In addition, we have also confirmed that another GCN2-RNAi (NIG #1609R-2) did not affect the p-eIF2α levels in *wol*$^{-/-}$ clones as well as their elimination (please see Fig. R7, below).

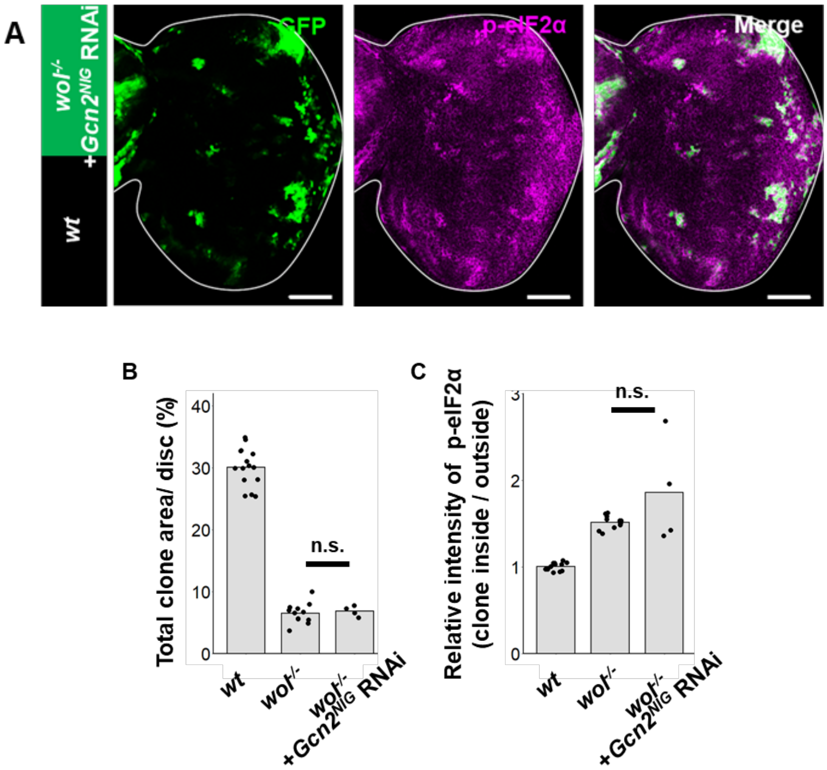

Fig. R7

(A) Eye disc bearing eyFLP-induced MARCM clones of wol$^{ccp\text{-}28\text{-}/\text{-}}$ + Gcn2-RNAi (NIG #1609R-2) cells stained with anti-phosphorylated eIF2α. (B and C) Quantification of the relative size of GFP-labeled clones or relative intensity of anti-phosphorylated eIF2α staining shown in A. Error bars, SD; ***$p<0.001$ by Welch's t-test.
Scale bars, 50µm.

Thus, we have now cited the Armstrong *et al*. paper in the revised manuscript as follows:

(page 6)

"These effects were not observed by knockdown of another eIF2α kinase GCN2 [26] (Fig. S2A), indicating that *wol* clones are eliminated via activation of the UPR pathway."

6. In Supp Fig. 3 the label Xbp1s-GFP should be corrected to Xbp1-GFP

Response:

We thank the reviewer for the comment. We have now corrected the label "Xbp1s-GFP" to "Xbp1-GFP" in the entire manuscript (please see page 5, page 6, Figure 1L' and M', and Suppl Fig 3A).
